# Modulation of immunological responses by aqueous extract of *Datura stramonium L.* seeds on cyclophosphamide-induced immunosuppression in Wistar rats

**DOI:** 10.1186/s12865-022-00519-y

**Published:** 2022-10-19

**Authors:** Parker Elijah Joshua, Junaidu Yahaya, Daniel Emmanuel Ekpo, Joyce Oloaigbe Ogidigo, Arome Solomon Odiba, Rita Onyekachukwu Asomadu, Samson Ayodeji Oka, Olasupo Stephen Adeniyi

**Affiliations:** 1 Department of Biochemistry, Faculty of Biological Sciences, University of Nigeria, 410001 Nsukka, Enugu State Federal Republic of Nigeria; 2 Department of Human Physiology, Faculty of Basic Medical Sciences, Colleges of Health Sciences, Kogi State University, P.M.B. 1008, Anyigba, Kogi State Federal Republic of Nigeria; 3 Bioresources Development Centre, National Biotechnology Development Agency (NABDA), Federal Capital Territory, Abuja, Federal Republic of Nigeria; 4 Department of Molecular Genetics and Biotechnology, Faculty of Biological Sciences, University of Nigeria, 410001 Nsukka, Enugu State Federal Republic of Nigeria; 5 Department of Biochemistry, College of Life Science and Technology, Guangxi University, Nanning, 530007 People’s Republic of China; 6 National Engineering Research Centre for Non-Food Biorefinery, Guangxi Academy of Sciences, Nanning, 530007 People’s Republic of China; 7 Department of Medical Biochemistry, Faculty of Basic Medical Sciences, Colleges of Health Sciences, Kogi State University, P.M.B. 1008, Anyigba, Kogi State Federal Republic of Nigeria; 8 Department of Physiology, Faculty of Basic and Allied Medical Sciences, Benue State University, Makurdi, Benue State Federal Republic of Nigeria

**Keywords:** Medicinal plants, *Datura stramonium* L., Immune system, Immunosuppression, Immunoglobulins, Cyclophosphamide, Wistar rats

## Abstract

**Background:**

*Datura stramonium L.* (Solanaceae) is used traditionally in west Africa to treat asthma, epilepsy, rheumatoid arthritis, filariasis microbial infections and conjunctivitis. This study investigated the immunomodulatory effects of aqueous seed extract of *D. stramonium L.* (ASEDS) on Wistar rats.

**Methods:**

Thirty Wistar albino rats (180–200 g) were randomized into 6 groups (n = 5). Group 1 received distilled water only. Rats in groups 2–6 were pretreated with 10 mg/kg body weight (b.w.) Cyclophosphamide orally for 27-days to induce immunosuppression. Thereafter, they received treatment orally for 28 days as follows: Group 2 (distilled water), group 3 (5 mg/kg b.w. Levamisole), groups 4–6 (60, 90 and 120 mg/kg b.w. ASEDS, respectively). HPLC was used to determine major compounds in ASEDS. The effects of ASEDS on immune cells, immunoglobulins A, G and M levels, lipoproteins, and antioxidant status of rats were evaluated.

**Results:**

ASEDS indicated high content of Acutumine, Quinine, Catechin, Chlorogenic acid, Gallic acid, Quercetin, Vanillic acid, Luteolin, Formosanin C, Saponin, Cyanidin, Tannic acid, 3-Carene, Limonene and α-terpineol. Cyclophosphamide triggered significant (*p* < 0.05) reduction in total leucocyte count and differentials, IgA, IgG, high-density lipoproteins (HDL), catalase, superoxide dismutase, glutathione peroxidase, vitamins A, C and E levels of untreated rats. Administration of ASEDS led to significant (*p* < 0.05) improvement in immune cell counts, immunoglobulin synthesis, high-density lipoprotein concentration, and antioxidant status of rats in the treated groups.

**Conclusions:**

The results obtained from the study showed the immunomodulatory activity of ASEDS, thereby indicating its potential in immunostimulatory drug discovery.

## Background

Immunosuppression is a reduction in the capacity of immune system to respond effectively to antigens including surface antigens on tumor cells. It can occur as a result of chronic infections, accumulation of toxic chemicals in the body and exposure to high doses of radiations [1]. According to Hutchinoson and Geissler [2] immunosuppression has been adopted deliberately as a means of treating autoimmune diseases and preventing acute graft rejection. Despite these benefits, prolonged immunosuppression would lead to infections, bone marrow suppression, cancer and infertility [3]. Cyclophosphamide is one of the potent immunosuppressive drugs belonging to the group known as oxazaphosporine [4], and has been widely investigated for its immunomodulatory effects [5]. The drug when ingested undergoes extensive metabolism in the liver via the cytochrome P-450 and produces phosphoramide as its active metabolite. Phosphoramide is an alkylating agent which inhibits DNA replication by irreversibly interacting with it at number seven atom of guanine base. This interaction causes cell death among resting and dividing leucocytes and thus leads to impair humoral and cellular immune responses [6].

Cyclophosphamide is a well-known chemotherapeutic and immune suppressive agent, and is widely in research to weaken the immune system of experimental animals [7]. The drug has been in clinical use for over 45 years, and is applied in the treatment of cancer and as an immunosuppressive agent for the treatment of autoimmune and immune-mediated diseases. The immunosuppressive as well as immunomodulatory properties of cyclophosphamide is well documented [7–9].

Immuno-modulators are substances that can either enhance or suppress any component of immune system. Jantan et al. [10] defined natural immuno-modulators as natural products of herbal origin called phytochemicals. Therefore, plant-derived substances can be used to modulate an immunosuppressed system and these substances could be present in the extract of *D. stramonium* seeds. Examples of immuno-modulators include adjuvants, vitamins, cytokines and herbs. Some vitamins have been widely reported to have immune-modulatory properties. Skrobot et al. [11] reported the immune-modulatory role of vitamin D in a detailed review, while Mousavi et al. [12] reported the immune-modulatory and antimicrobial effects of vitamin C. Cytokines, some of which include; TNF, IL-1, IL-2, IL-4, IL-12, chemokines, IFN-ɤ are a group of low-molecular-weight regulatory proteins secreted by the leukocytes and other cells in the body. They play important role in immune and inflammatory response, particularly interferon (IFN)-γ, tumor necrosis factor (TNF)-α, and interleukin (IL)-1β [13, 14]. Certain herbs are also known to have immunomodulatory properties. Some of these include; *Coriolus versicolor* [15], Neem plant, ginger, *Ponax ginseng, and Withania somnifera* [16].

Medicinal plants are known to have great potential for treatment and management of certain diseases including those affecting different components of the immune system [17]. One of such plant is *Datura Stramonium L.* (Solanaceae), an annual plant that is native to Asia and Africa [18]. In Nigeria especially in Kogi State, it is found growing in abandoned farmlands and dumpsites. It is popularly known for its narcotic effect as a result called names such as Devil’s apple, Angel’s trumpet and Jimson weed but its indigenous names include Jegemi in Igala, Myaramwo in Igbo, Gegemu in Yoruba and Zakami in Hausa tribes of Nigeria. It is used traditionally to treat asthma, epilepsy, rheumatoid arthritis, filariasis and as antimicrobials, notably against *Staphylococcus aureus, Aspergillus niger* and conjunctival virus [19].

Rapid advances in human civilization have led to the increasing presence of various synthetic chemicals in the environment. Some of these chemicals and their products are immunotoxins which are capable of rendering hosts more susceptible to infectious diseases as a result of immunosuppression [20]. According to the World Health Organization (WHO), most antibiotics would lose their antimicrobial action by the year, 2020 [21]. Consequently, immunomodulation becomes a reliable alternative in the treatment of diseases particularly those that are immune-mediated since its mechanism can enhance both specific and non-specific immunities [22].

The toxicity of *D. stramonium* L is well documented [23–26]. Ogunmoyole et al. [26], and Joshua et al. [27] reported the multi-organ toxicity of *Datura stramonium* seed extracts. These studies investigated acute and subacute toxicities, as well as toxicities to liver, kidney, brain, heart tissues, and peroxidation of lipids. At the moment a dearth of information still exist as regards the effect of *D. stramonium* on the immune system. It therefor becomes imperative for us to investigate the effects of the seed extract of *D. stramonium* of some immune system indicators and cells.

## Results

### Percentage yield of ASEDS

Aqueous crude extraction of 257.6 g of powdered *D. stramonium L.* seeds yielded 20.35 g of ASEDS, representing 7.9% of the total quantity of the powdered plant material extracted.

### Preliminary Phytochemical composition of ASEDS

Qualitative phytochemical screening of ASEDS indicated high content of phenols, alkaloids, and flavonoids, with moderate contents of glycosides, terpenoids and carbohydrates, whereas saponins, tannins and steroids were present in lower quantities Table 1.

**Table 1:** Phytochemical composition of ASEDS

Phytochemicals	Qualitative analysis (bioavailability)	Quantitative analysis (mg/g)
Alkaloids	+++	269.05 ± 1.63
Carbohydrates	++	52.59 ± 0.59
Flavonoids	+++	278.65 ± 0.63
Phenols	+++	396.06 ± 6.56
Glycosides	++	148.71 ± 1.83
Saponins	+	0.76 ± 0.07
Steroids	+	2.06 ± 0.10
Tannins	+	0.03 ± 0.00
Terpenoids	++	72.04 ± 21.08

Results are expressed as mean ± standard deviation, n = 3. Highly present (+++), moderately present (++), scanty (+). ASEDS: Aqueous seed extract of *Datura stramonium*

**Fig. 1:**
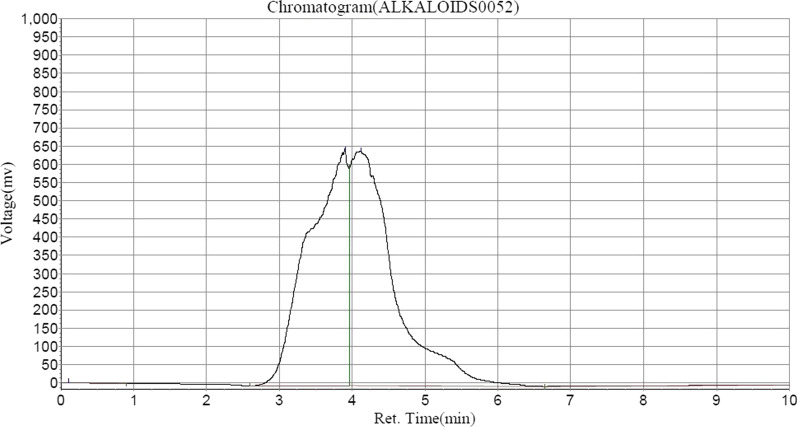
HPLC–UV chromatogram of alkaloids in ASEDS

**Fig. 2:**
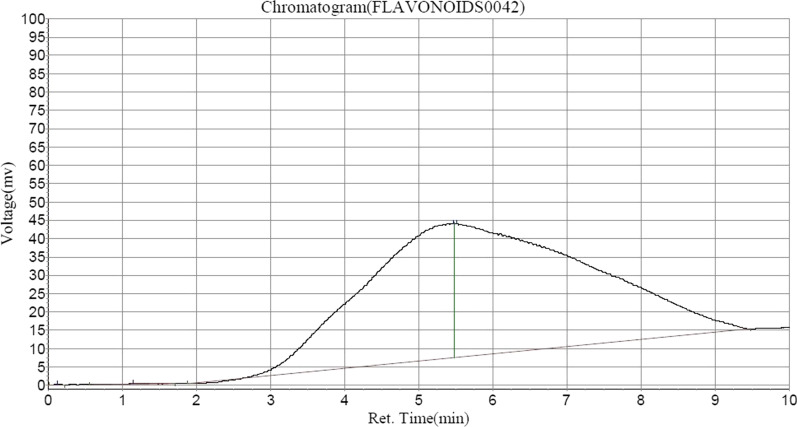
HPLC–UV chromatogram of flavonoids in ASEDS

**Fig. 3:**
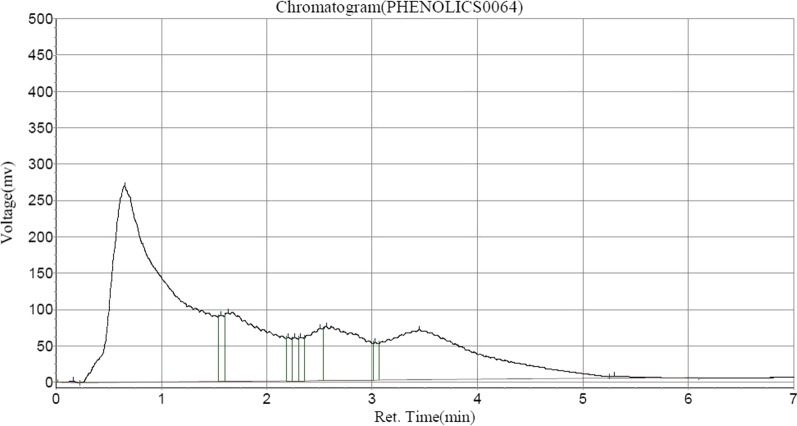
HPLC–UV chromatogram of phenolic compounds in ASEDS

Following HPLC analysis of ASEDS (Table 2) shows the presence of alkaloids such as, quinine in highest concentration, followed by Acutumine and Yohimbine respectively (Fig. 1). Catechin and chlorogenic acids were the dominant flavonoids identified (Figs. 2, 3, Table 3).

**Table 2:** Alkaloid composition of aqueous seed extract of *Datura stramonium*

Peak no	Peak ID	Ret. time	Height	Area	Conc (mg/100 g)
1	Solvent font	0.107	687.514	7728.350	0.0151
2	Acutumine	3.907	649,950.750	24,404,468.000	47.6634
3	Quinine	4.115	643,816.625	26,735,024.000	52.2151
4	Yohimbine	10.048	47.022	54,515.801	0.1065

**Table 3:** Flavonoid composition of aqueous seed extract of *Datura stramonium*

Peak no	Peak ID	Ret. time	Height	Area	Conc (mg/100 g)
1	Solvent font	0.123	136.000	626.150	0.0081
2	Sinapic acid	1.148	123.360	3229.300	0.0419
3	Chlorogenic acid	5.465	36,552.203	3,154,933.250	40.9552
4	Catechin	5.515	36,395.387	4,544,595.000	58.9948

A vast majority of phytochemicals in ASEDS were phenolic compounds including; gallic acid which was identified in highest quantity, quercetin, vanillic acid, luteolin, benzoic acid, syringic acid, ferulic acid, ellagic acid, p-coumaric acid, and apigenin (Table 4).

**Table 4:** Phenolic composition of aqueous seed extract of *Datura stramonium*

Peak no.	Peak ID	Ret. time	Height	Area	Conc (mg/100 g)
1	Solvent font	0.165	2548.520	10,710.100	0.0519
2	Gallic acid	0.648	269,477.750	9,916,016.000	48.0627
3	Syringic acid	1.565	91,027.008	316,475.156	1.5339
4	Vanillic acid	1.632	94,237.773	2,668,614.000	12.9347
5	Ellagic acid	2.207	59,821.496	205,782.844	0.9974
6	*p*-coumaric acid	2.265	59,539.539	205,195.594	0.9946
7	Ferulic acid	2.323	59,989.582	207,343.656	1.0050
8	Benzoic acid	2.507	72,019.438	697,476.750	3.3807
9	Luteolin	2.565	74,121.477	1,840,839.750	8.9225
10	Unidentified	3.032	51,701.832	178,699.016	0.8661
11	Quercetin	3.448	68,558.859	4,333,013.500	21.0020
12	Apigenin	5.298	2953.826	51,254.898	0.2484

**Fig. 4:**
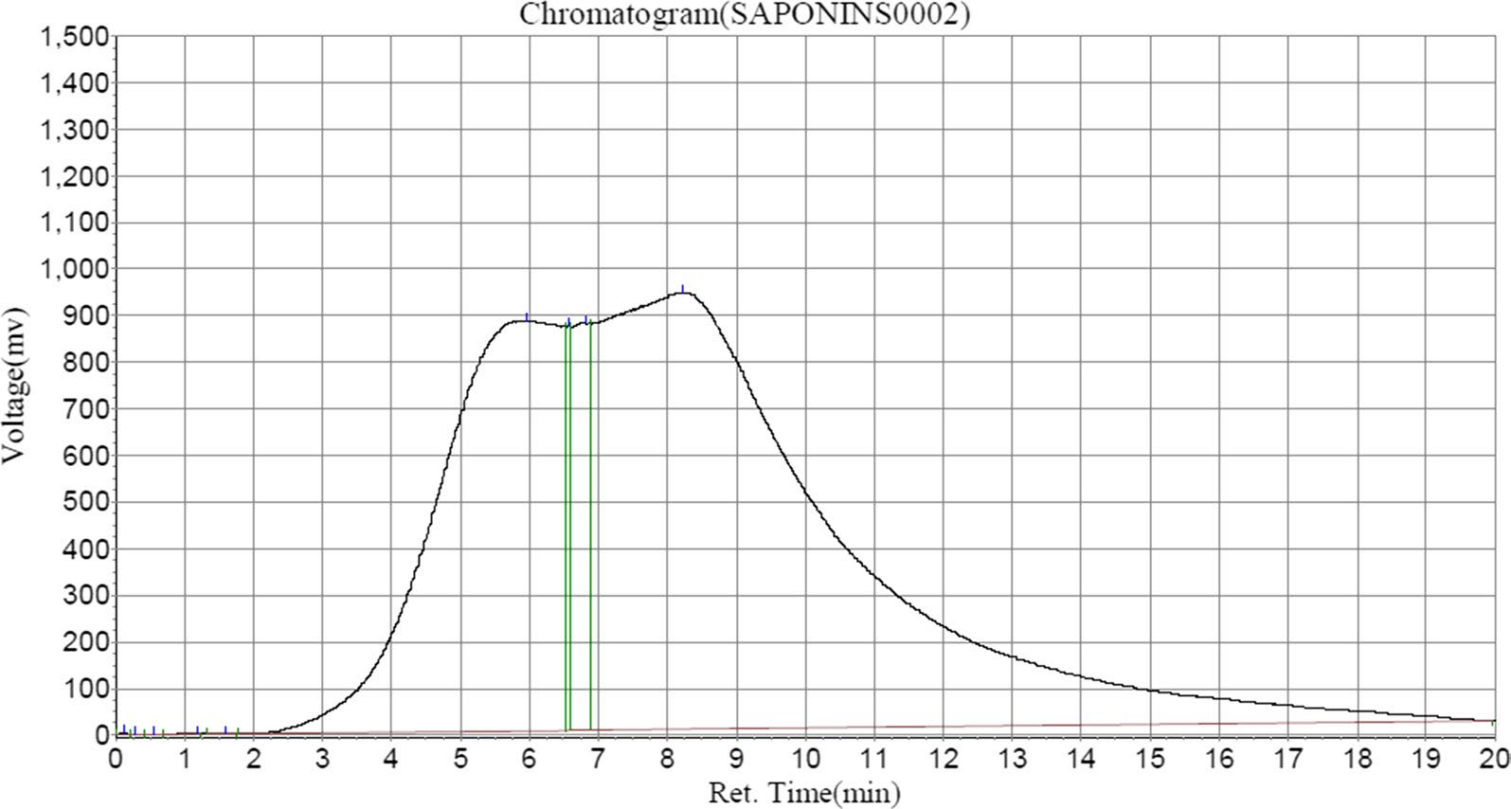
HPLC–UV chromatogram of saponins in AESDS

**Fig. 5:**
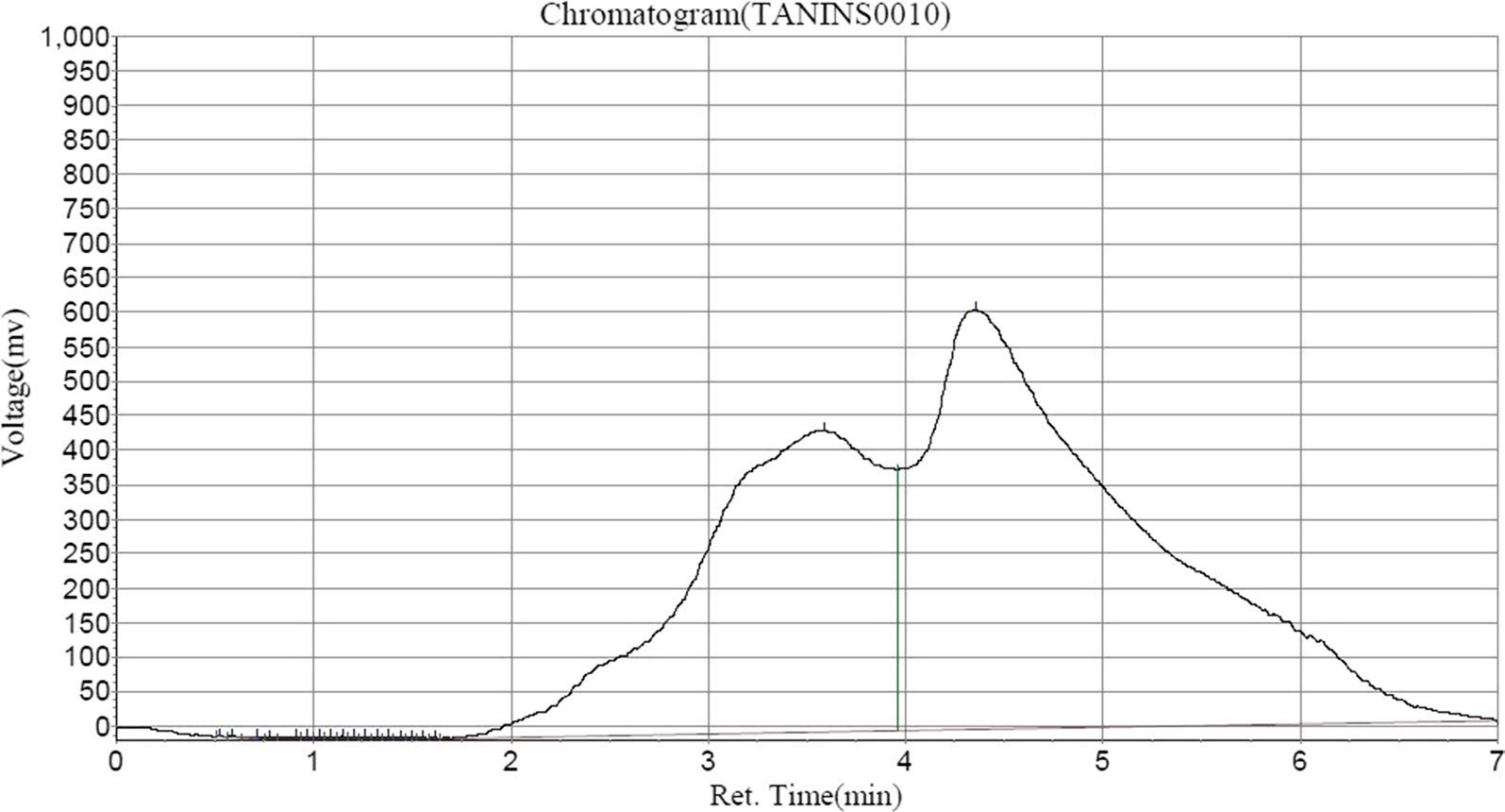
HPLC–UV chromatogram of tannins in ASEDS

**Fig. 6:**
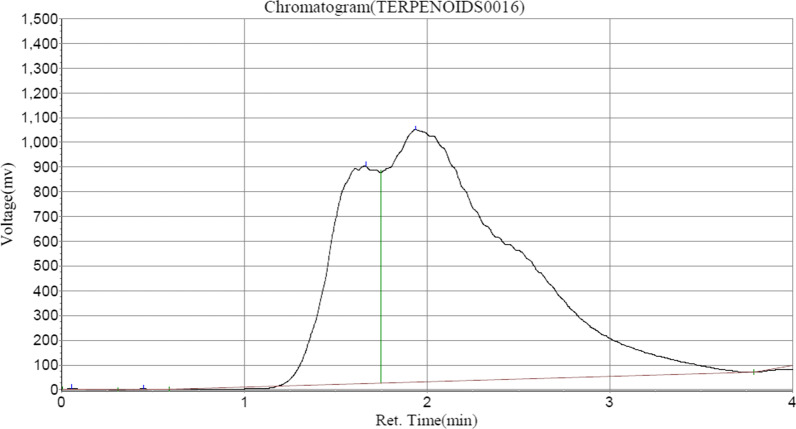
HPLC–UV chromatogram of terpenoids in ASEDS

Saponin and Formasanin C formed the major contents of saponins in ASEDS (Table 5, Fig. 4), whereas tannic acid and cyanidin constituted a vast majority of tannins identified in ASEDS by HPLC (Table 6, Fig. 5). The results also showed higher concentration of α-terpineol followed by limonene, 3-carene and α-pinene respectively (Fig. 6, Table 7).

**Table 5:** Saponin composition of ASEDS

Peak no.	Peak ID	Ret. time	Height	Area	Conc (mg/100 g)
1	Solvent font	0.123	3280.923	18,391.600	0.0052
2	Unidentified	0.273	284.348	1629.350	0.0005
3	Unidentified	0.548	929.680	7700.036	0.0022
4	Unidentified	1.190	87.330	10,357.414	0.0029
5	Unidentified	1.590	80.615	1044.800	0.0003
6	Formosanin C	5.948	880,484.250	110,008,360.000	31.0704
7	Unidentified	6.565	868,560.813	3,469,736.000	0.9800
8	Unidentified	6.815	874,428.313	15,676,592.000	4.4276
9	Saponin	8.215	937,310.750	224,868,064.000	63.5109

**Table 6:** Tannin composition of aqueous seed extract of *Datura stramonium*

Peak no.	Peak ID	Ret. time	Height	Area	Conc (mg/100 g)
1	Unidentified	0.523	1311.143	2185.250	0.0029
2	Unidentified	0.590	2583.000	6291.150	0.0083
3	Unidentified	0.715	1621.000	6206.000	0.0082
4	Unidentified	0.782	1121.651	2753.759	0.0036
5	Unidentified	0.915	2372.123	12,199.299	0.0161
6	Unidentified	0.973	2719.642	7987.166	0.0105
7	Unidentified	1.032	2871.160	2871.160	0.0112
8	Unidentified	1.090	2896.679	8677.396	0.0114
9	Epicatechin	1.148	3018.198	8872.215	0.0117
10	Unidentified	1.207	2900.717	8468.930	0.0112
11	Unidentified	1.265	2738.236	7893.744	0.0104
12	Unidentified	1.323	2498.755	7120.963	0.0094
13	Unidentified	1.382	2195.274	6140.978	0.0081
14	Unidentified	1.440	1855.792	5030.093	0.0066
15	Unidentified	1.498	1490.311	3834.611	0.0051
16	Unidentified	1.557	1162.830	2742.026	0.0036
17	Unidentified	1.615	1006.349	2227.541	0.0029
18	Cyanidin	3.590	438,822.344	29,362,538.000	38.7257
19	Tannic acid	4.357	609,192.188	46,352,232.000	61.1330

**Table 7:** Terpenoid composition of aqueous seed extract of *Datura stramonium*

Peak no.	Peak ID	Ret. time	Height	Area	Conc (mg/100 g)
1	Solvent font	0.057	4872.167	15,119.901	0.0051
2	α-Pinene	0.448	2675.000	15,923.700	0.0054
3	3-Carene	1.665	880,226.000	5.5048	5.5048
4	Limonene	1.940	1,021,213.250	48,736,156.000	16.4145
5	α-terpineol	8.948	593,944.000	231,797,312.000	78.0703

### Effect of ASEDS on immune cells of cyclophosphamide-induced immunosuppression in rats

Table 8 shows the Total leucocyte count of the Negative control (group 2) to be significantly (*p* < 0.05) lower when compared to normal control and ASEDS treated groups. The normal control rats recorded significantly (*p* < 0.05) higher lymphocyte counts when compared to the negative control and the treated groups. Dose-dependent increases in leucocytes and lymphocytes count were observed in the ASEDS treat groups. We also observed a significant (*p* < 0.05) increase in neutrophils for the negative control relative to the normal control and the treated groups respectively. Immunosuppression also led to significant (*p* < 0.05) declines in monocytes and eosinophils of the negative control when compared to the normal control. Treatment with ASEDS and standard drug (Levamisole) improved the levels of monocytes and eosinophils in the test groups. Basophils where not present in the negative control rat group following cyclophosphamide pretreatment. However, treatment with ASEDS and Levamisole triggered significant (*p* < 0.05) increase in basophil count of the test groups.

**Table 8:** Effect of ASEDS on immune cells of cyclophosphamide-induced immunosuppression in rats

Groups	Total leukocyte count (× 10^9^/L)	Differential cell count (%)				
		Lymphocytes (%)	Neutrophils (%)	Monocytes (%)	Eosinophils (%)	Basophils (%)
1	9.98 ± 1.05^a^	75.88 ± 1.94^a^	18.02 ± 1.00^a^	3.38 ± 1.87^a^	2.12 ± 0.87^a^	0.60 ± 0.54^a^
2	2.30 ± 1.05^b^	61.60 ± 3.78^b^	34.00 ± 3.39^b^	2.20 ± 0.83^b^	2.20 ± 0.83^a^	0.00 ± 0.00^a^
3	9.90 ± 1.56^a^	31.60 ± 2.83^c^	31.40 ± 1.14^b,c^	13.40 ± 1.14^c^	22.80 ± 2.68^b^	1.40 ± 0.54^b^
4	16.12 ± 0.53^c^	29.20 ± 4.81^c^	57.20 ± 2.65^d^	9.60 ± 2.60^d^	2.40 ± 1.14^a^	1.00 ± 1.00^b^
5	18.74 ± 2.75^c,d^	38.00 ± 6.20^d^	39.20 ± 0.83^e^	8.48 ± 0.50^d^	2.36 ± 1.46^a^	0.40 ± 0.89^c^
6	21.40 ± 1.95^d^	55.86 ± 2.89^e^	28.86 ± 5.09^c^	17.04 ± 22.91^e^	7.24 ± 2.83^d^	1.00 ± 0.00^b^

Results are expressed as mean ± standard deviation, n = 5. Mean values with different lowercase alphabets as superscripts when compared down the groups are significantly different at *p* < 0.05. ASEDS: Aqueous seed extract of *Datura stramonium*. 1: Normal control; 2: Negative control; 3: Positive control; 4: Immunosuppressed + 60 mg/kg of extract; 5: Immunosuppressed + 90 mg/kg of extract; 6: Immunosuppressed + 120 mg/kg of extract

### Effect of ASEDS on immunoglobulin A, G and M levels of cyclophosphamide-induced immunosuppression in rats

**Fig. 7:**
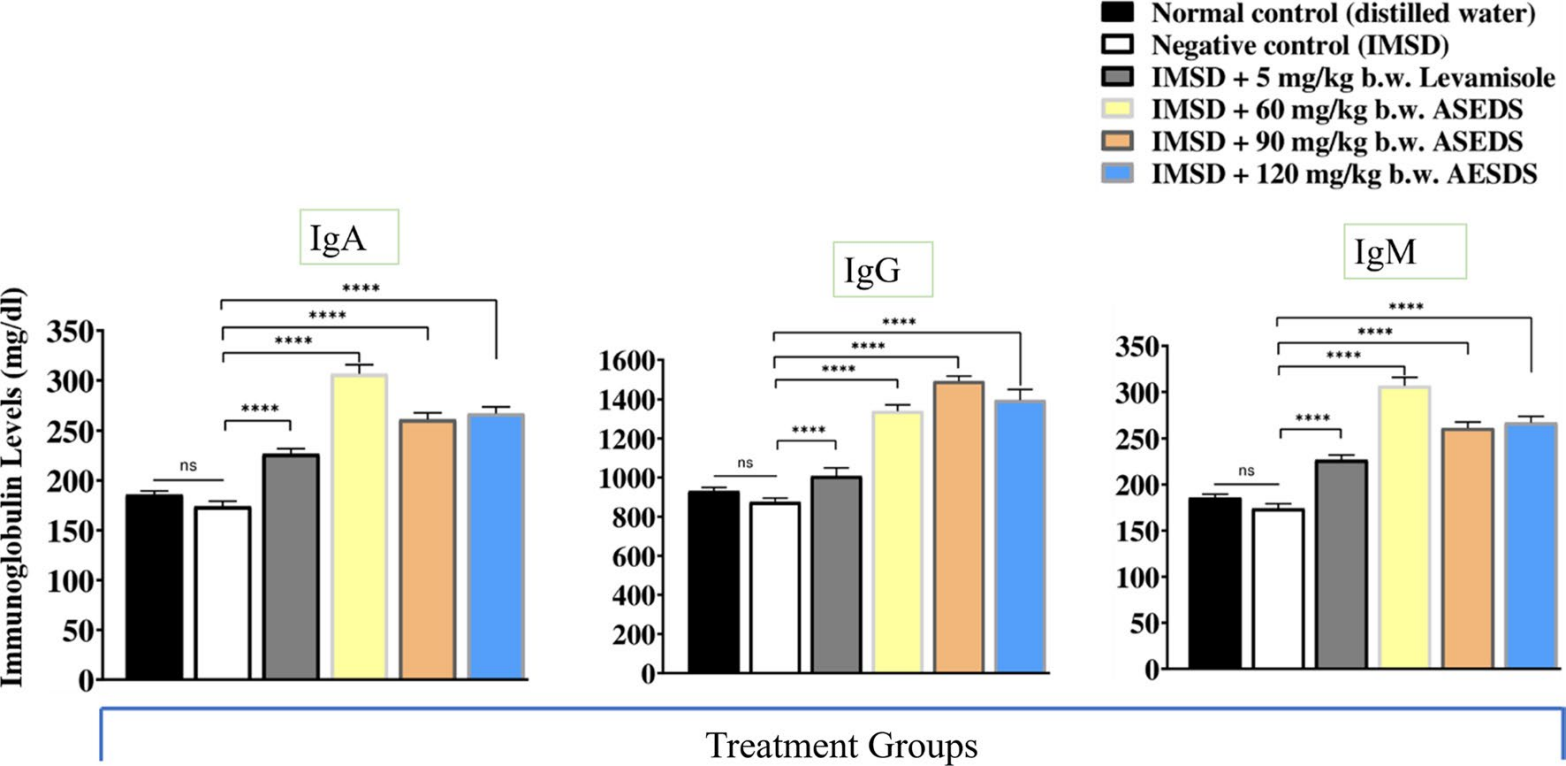
Effect of ASEDS on immunoglobulin A, G and M levels of cyclophosphamide-induced immunosuppression in rats. Mean values with **** is significantly (*p* < 0.0001) different relative to the value obtained by the negative control rats. *ASEDS* Aqueous seed extract of *Datura stramonium*, *IgA* Immunoglobulin A, *IgG* Immunoglobulin G, *IgM* Immunoglobulin M, *IMSD* Immunosuppressed, *ns* no significant difference

The effect of ASEDS on immunoglobulins A, G, and M levels of cyclophosphamide-immunosuppressed rats is shown in Fig. 7. The results indicated significant (*p* < 0.05) declines in IgA, IgG, and IgM levels of the negative control relative to the normal control rats. Treatment with graded doses of ASEDS resulted in significant (*p* < 0.05) elevation in IgA, IgM and IgG levels with group 4 rats, treated with 60 mg/kg b.w. ASEDS registering the highest levels of IgA (306.63 ± 9.23 mg/dl). Group 5 rats, treated with 90 mg/kg b.w. ASEDS recorded highest levels of IgG (259.94 ± 31.68 mg/dl) and IgM (1491.16 ± 26.35 mg/dl) synthesis post-ASEDS treatment.

### Effect of ASEDS on the activities of antioxidant enzymes of cyclophosphamide-induced immunosuppression in rats

**Fig. 8:**
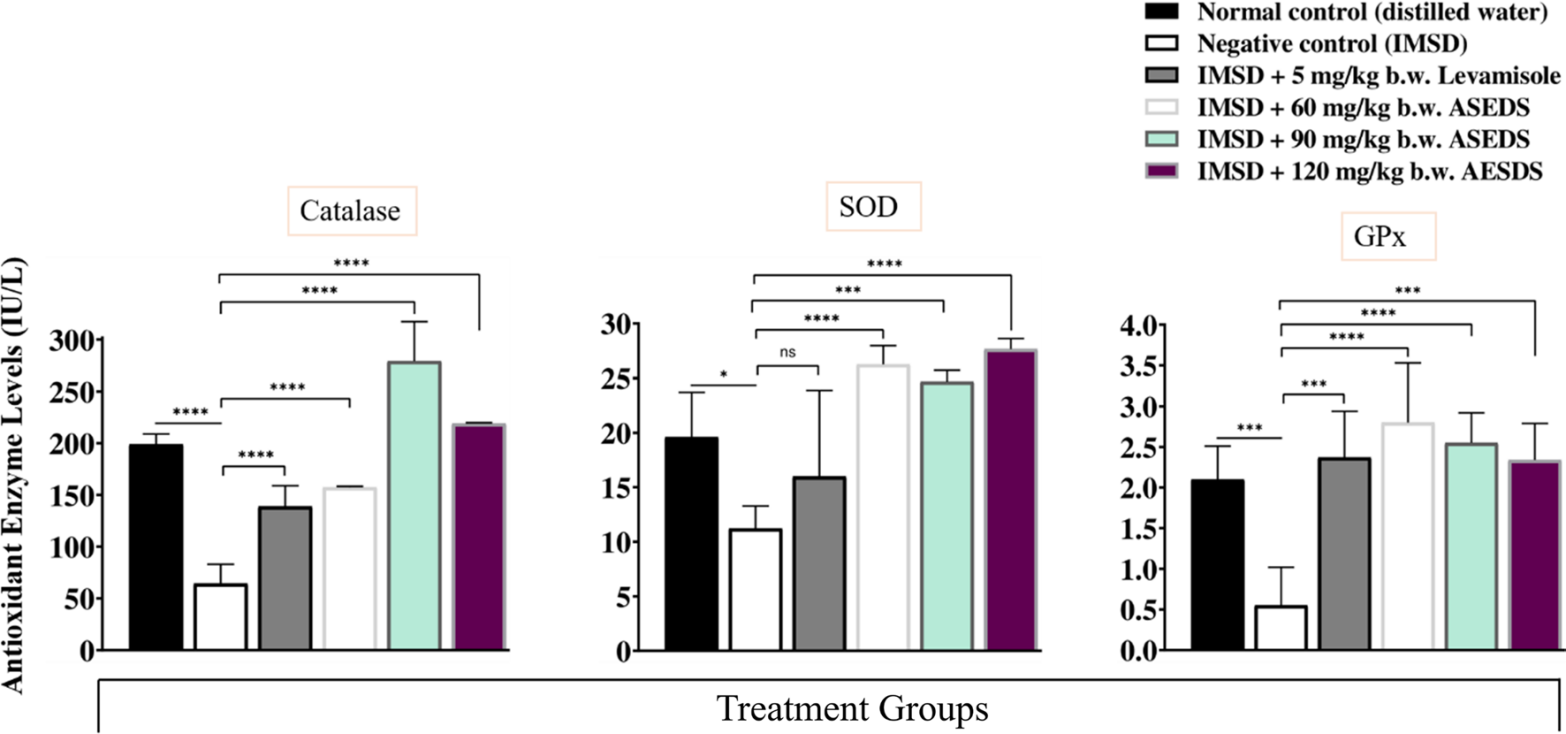
Effect of ASEDS on the activities of antioxidant enzymes of cyclophosphamide-induced immunosuppression in rats. Mean values with * (*p* < 0.05), *** (*p* < 0.001), or **** (*p* < 0.0001), is significantly different relative to the value obtained by the negative control rats. *ASEDS* Aqueous seed extract of *Datura stramonium*, *SOD* Superoxide dismutase, *GPx* Glutathione peroxidase, *IMSD* Immunosuppressed, *Ns* no significant difference

Figure 8 shows the effect of ASEDS on the activities of antioxidant enzymes of cyclophosphamide-induced immunosuppression in rats. From the data obtained, we observed significant (*p* < 0.05) declines in the activities of serum CAT, SOD and GP_X_ following immunosuppression. However, treatment with graded doses of ASEDS led to significant (*p* < 0.05) elevations in the activities of these antioxidant enzymes in all test groups when compared to the negative control.

### Effect of ASEDS on antioxidant vitamin concentrations of cyclophosphamide-induced immunosuppression in rats

**Fig. 9:**
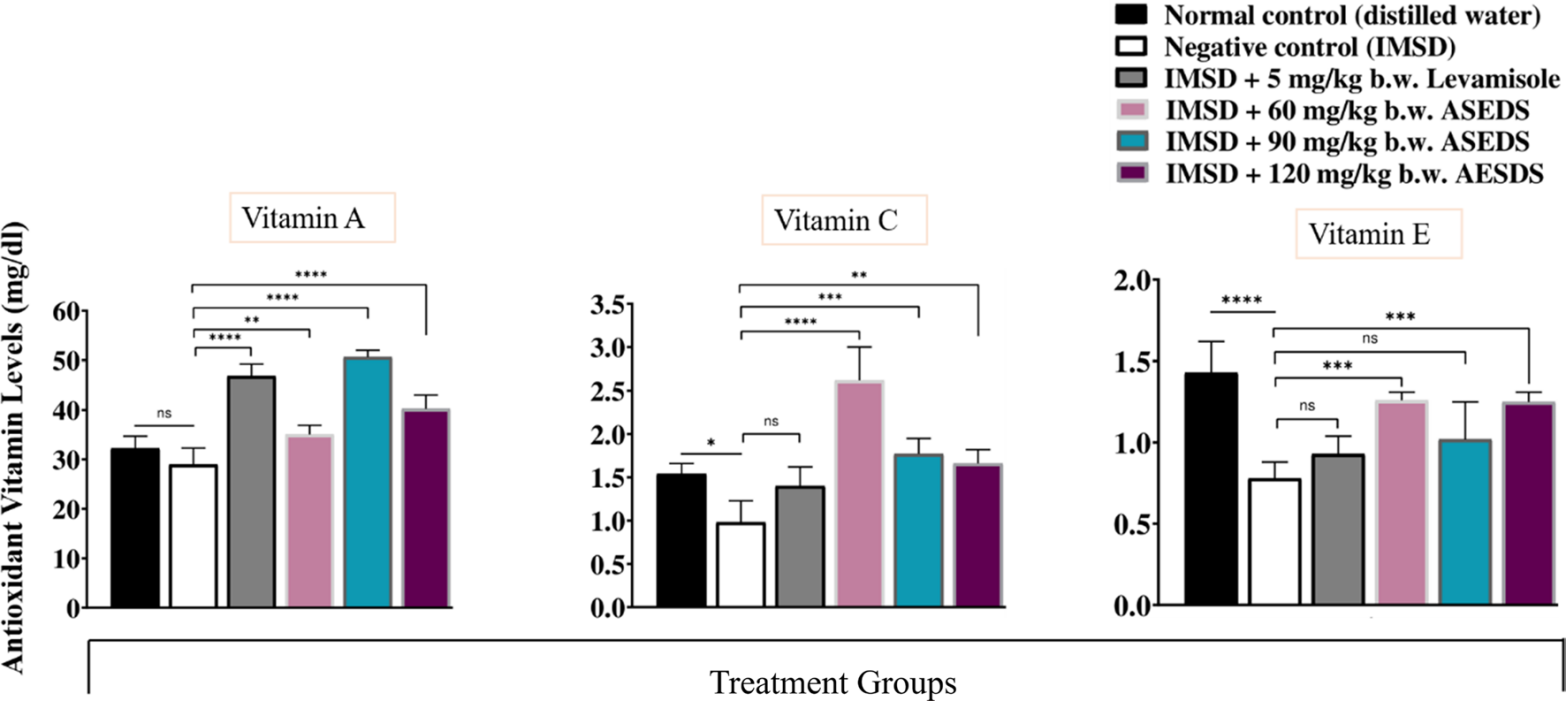
Effect of ASEDS on antioxidant vitamin concentrations of cyclophosphamide induced immunosuppression in rats. Mean values with *(*p* < 0.05), ** (*p* < 0.01), *** (*p* < 0.001), or **** (*p* < 0.0001), is significantly different relative to the value obtained by the negative control rats. *ASEDS* Aqueous seed extract of *Datura stramonium*, *IMSD* immunosuppressed, *Ns* no significant difference

The effect of ASEDS on antioxidant vitamin concentrations of cyclophosphamide-induced immunosuppression in rats is shown in Fig. 9. The results indicated significantly (P < 0.05) lover concentrations of vitamins A, C and E for the negative control following cyclophosphamide pre-treatment, relative to the normal control. Following administration of ASEDS at doses of 60, 90 and 120 mg/kg b.w., we observed significantly (P < 0.05) higher concentrations of the antioxidant vitamins in all ASEDS-treated groups and standard control relative to the negative control.

### Effect of ASEDS on lipoprotein levels of cyclophosphamide-induced immunosuppression in rats

**Fig. 10:**
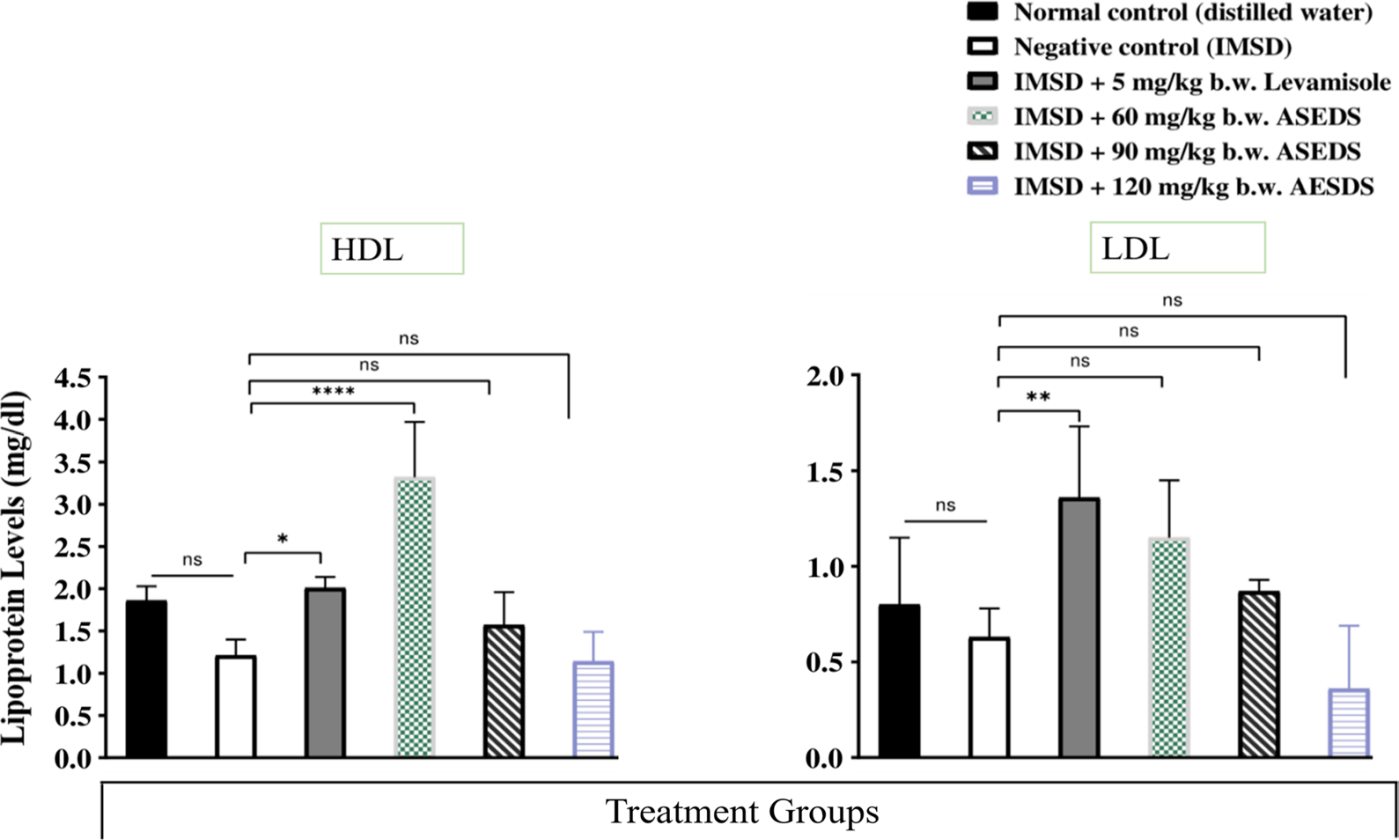
Effect of ASEDS on lipoprotein levels of cyclophosphamide-induced immunosuppression in rats. Mean values with * (*p* < 0.05), ** (*p* < 0.01), or **** (*p* < 0.0001), is significantly different relative to the value obtained by the negative control rats. *ASEDS* Aqueous seed extract of *Datura stramonium*, *HDL* high-density lipoprotein, *LDL* low-density lipoprotein, *ns* no significant difference

Figure 10 presents the effect of ASEDS on lipoprotein levels of cyclophosphamide-induced immunosuppression in rats. Down the groups, the results indicated significant (*p* < 0.05) increases in mean serum LDL and HDL concentrations of Groups 3, 4 and 5 rats when compared to the normal control and negative control. Across the groups, we observed significant (*p* < 0.05) increases in HDL concentration when compared to the corresponding LDL concentration.

## Discussion

The use of plant extracts in traditional medicine continues to provide therapeutic needs of greater percentage of world’s population especially in developing countries [28, 29]. *D. stramonium* is one of such valuable plants with rich therapeutic properties, and has been subjected to myriads of uses and applications due to the presence of secondary metabolites the plant contains [30]. In the present study, the efficacy of ASEDS in modulation the effect of cyclophosphamide-induced immunosuppression in Wistar rats was assessed. According to Farombi et al. [31] several plant-derived compounds have been identified over the years for their immunomodulatory characteristics. As a result, numerous illnesses can be alternatively treated via immunomodulation.

Preliminary qualitative phytochemical screening of ASEDS showed the presence of alkaloids, flavonoids, saponins, phenols, glycosides, tannins, terpenoids, steroids and carbohydrates. This agrees largely with the findings of Bano and Adeyemo [32] who reported that the ethanol seed extract of *D. stramonium* contains saponins, flavonoids, terpenoids, steroids, carbohydrates, alkaloids, phenols, tannins, fats and oils. The presence of these phytochemical constituents could be attributed to the geographical location where the plant material was collected, and solvent used for the extraction of the plant materials [33]. Quantitatively, phenols, alkaloids and flavonoids were of the highest concentration, while the least was tannins, steroids and saponins (Table 1). This conforms to the results obtained by Sharma and Sharma [34] in terms of degree of presence of these phytochemicals where they reported high presence of phenols, moderate contents of alkaloids, flavonoids, terpenoids, glycosides and soluble carbohydrates, whereas steroids, saponins and tannins were present in low quantities. Some of these phytochemical constituents have been reported to exhibit stimulatory or inhibitory effect on various components of immune system. The antioxidant role of phenolic and flavonoids can confer anticancer property of the extract on the animals [30]. Tannin was reported by Haslam [35] to stimulate phagocytosis in macrophages and dendritic cells hence the extract could participate in both innate and adaptive immune responses. The relative high presence of alkaloids can confer antitumor activity and enhance immune response [36]. Dash et al. [37] stated the role of flavonoids increasing the activity of helper T-cells, cytokines (interleukin-2 and g-interferon), and macrophages, indicating their usefulness in the treatment of several diseases caused by immune dysfunction. According to Jorrossay and Thelen [38], steroids were the most important phytochemical constituent that can reinstate a balance the Th1 and Th2 cells, thus can determine the type of immune response of a system. Carbohydrates could also contribute the immunologic capability of the system [39], by enhancing the recognition of antigens when they presented. Terpenoids were reported to possess ability to modulate critical signaling pathways such as nuclear transcription factor kappa B which helps in prompt synthesis of cytokines [40].

The determination of total leucocyte and differential count are important markers of immune function. In this study, the total leukocyte counts of the ASED-treated groups significantly (*p* < 0.05) increased relative to the negative control (Table 8). This effect could be mediated by cytokines such as colony stimulating factor (CSF) 2, a monomeric glycoprotein secreted by macrophages, T-cells, mast cells, natural killer cells, endothelial cells and fibroblasts in response to the presence of xenobiotic. CSF 2 triggers proliferation of leucocytes by activation of hematopoietic stem cells (HSCs). This shows the ability of ASEDS to stimulate leukocytosis. The result is consistent with the findings of Fatoba et al. [41] who stated that bucks treated with aqueous seed extract of *D. stramonium* recorded higher white blood cell counts than the control except at the highest extract dose. There was a significant (*p* < 0.05) decrease in total leucocyte count in the negative control relative to the standard and normal controls, thus validating the efficacy of cyclophosphamide has an immunosuppressive drug. Phosphoramide, an alkylating agent generated by the bio-activation of cyclophosphamide could be responsible for the death of leucocytes as observed in the negative control [42]. Leucocyte count of the standard control was observed to be similar to the that of the normal control which demonstrated efficacy of Levamisole as an immune-booster [43]. This agrees with finding of Undiandeye et al. [44] who reported an increase in leucocyte count following treatment of an immunocompromised goat. The increase in leucocyte counts increases the ability of the host to defend the body against invasion of foreign agents [45].

The lymphocyte counts of Groups 5 and 6 was observed to be significantly (*p* < 0.05) higher when compared to the standard control. It was observed that the effect of ASEDS on lymphocyte counts was dose-dependent (Table 8). This implies that extract has capacity to boost both antibody-dependent and cell-mediated immune responses since lymphocytes play dominate role in immune responses. However, the result is contrary to the findings of [41] who reported increase in lymphocyte counts with decreasing extract dosage. Neutrophil counts increased significantly (*p* < 0.05) in Group 4 compared to the control groups and other test groups; suggesting that the extract can stimulate cell mediated elimination of bacterial pathogens because major phagocytes mobilized during bacterial invasion are neutrophils. As observed, the effect had an inverse dose dependence on other test groups. In other words, low doses of the extract appeared to stimulate more synthesis of neutrophils than high doses. This also disagrees with the findings of Fatoba et al. [41] where high extract doses stimulated increase in the count of neutrophils. Monocyte counts in group 6 increased significantly (*p* < 0.05) compared to all control groups. The extract therefore can enhance cell-mediated immune responses via phagocytosis and antigen presentation. Monocyte can differentiate into active macrophages and dendritic cells [46]. Eosinophils count increased significantly (*p* < 0.05) in Group 5 compared to normal and negative controls respectively. However, the eosinophil counts in the test groups showed significantly (*p* < 0.05) decreased relative to the standard control. This implies that the mechanism by which Levamisole could have stimulated leucocyte differentiation to optimize eosinophil levels. This corroborates the findings of Undiandeye et al. [44] in which eosinophils count of the goat treated with Levamisole increased significantly compared with the normal control.

Sari et al. [47] attributed the heightened immunomodulatory action of areca nut extract to the presence of catechin and quercetin also identified in ASEDS, which increased the concentration of WBCs and improved the activity and capability of macrophages in rats infected with S*taphylococcus aureus*.

Polyphenols are known to regulate immunity via interference with immune cell regulation, proinflammatory cytokines' synthesis, and gene expression. They inactivate NF-κB, MAPk and arachidonic acids pathways. These compounds also inhibit PI3K/AkT, IKK/JNK, mTORC1 and JAK/STAT pathways [48]. The modulation of inflammatory cytokines remains one of many common mechanisms by which polyphenols exert their immunomodulatory effects. Polyphenols such as quercetin and catechins found in ASEDS (Table 4, Fig. 3) exert their immunomodulatory effects on the balance between pro- and anti-inflammatory cytokines production. They enhance IL-10 release while inhibiting TNFα and IL-1β [49].

Kilani-Jaziri et al. [50], discovered that luteolin and apigenin, which were also found in ASEDS, significantly boosted humoral immune responses and increased lipopolysaccharide-stimulated splenocyte proliferation. Additionally, they markedly increased the activity of isolated murine splenocytes' cytotoxic T lymphocytes (CTL) and natural killer (NK) cells. Cardenas et al. [51] reported that Apigenin significantly modulated NF-κB activity in the lungs, indicating the ability of Apigenin to exert immune-regulatory activity in an organ-specific manner.

The immunomodulatory activity of gallic acid was reported by Shruthi et al., [52] as it improved antibody titer values and hematological indices of cyclophosphamide and cisplatin-induced immunosuppression in Swiss albino mice. Oral administration of Ferrulic acid reduced the levels of OVA-specific immunoglobulin E (IgE) and IgG1 and enhanced IgG2a antibody production in mice serum [53].

Kilani-Jaziri et al. [54], reported that phenolic acids such as caffeic, ferulic, and p-coumaric acids also identified in ASEDS significantly enhanced the killing activity of isolated NK and CTL cells. Their report showed that the phenolic acids exhibited an immunomodulatory effect which could be ascribed to their cytoprotective effect via their antioxidant capacity. In another study, Singh et al. [55] documented the immunomodulatory potential of ferulic acid via carbon clearance test, delayed type hypersensitivity reaction, neutrophils adhesion test, effect on serum immunoglobulins and cyclophosphamide induced neutropenia.

Immunoglobulins A, G and M are useful markers of immune response. Following cyclophosphamide-induced immunosuppression in rats, we observed significant (*p* < 0.05) decreases in the levels of immunoglobulins isotypes (IgA, IgG and IgM) of the negative control. Treatment with ASEDS resulted in significant (*p* < 0.05) elevations in IgA, IgG and IgM levels (Fig. 7). This effect could due to the ability of ASEDS to stimulate biosynthesis of interleukin-6 by T helper 2 cells which enhances B-lymphocytes differentiation into mature plasma cells that secret immunoglobulins. Thus, suggesting the potency of ASEDS to boost humoral immune responses [56]. Also observed was the fact that IgG level was highest among the determined immunoglobulin isotypes implying that ASEDS could enhance antibody dependent cytotoxicity [57]. A study by Bachhav et al. [58] showed that the alkaloid fraction of *Trichopus zeylanicus* stimulate defense system by modulating several immunological parameters. Analysis of ASEDS by HPLC indicated presence of alkaloids such as acutumine, quinine, and yohimbine (Table 2, Fig. 1).

Aqueous seed extract of *D. Stramonium L.* has been previously reported to restore altered antioxidant enzyme activities following cyclophosphamide-induced oxidative stress in rats [27]. In the present study, induction of immunosuppression following pre-treatment with cyclophosphamide led to significant (*p* < 0.05) declines in the activities of SOD, CAT and GP_X_ (Fig. 8). Administration of ASEDS resulted in significant (*p* < 0.05) elevation of the antioxidant enzymes activities thereby restoring the normal antioxidant balance, thus, suggesting the ability of ASEDS to boost the first line of defense against reactive oxygen species (ROS) generated from the bio-activation of cyclophosphamide. The results disagree with the report of Ogunmoyole et al. [26] who demonstrated that the activities of SOD, CAT and GP_X_ were depleted by administration of seed extract of *D. stramonium L*. regardless of the solvent used for the extraction. Cyclophosphamide induction also resulted in significant (*p* < 0.05) decrease in the concentrations of vitamins A, C, and E (Fig. 9). This could be accrued to the increased levels of free radicals generated as a result of lipid peroxidation induced by acrolein [27]. The decrease in vitamins A, C and E concentration is consistent with the work of Joshua et al. [27]. Treatment with graded doses of ASEDS significantly (*p* < 0.05) increased the concentration of these antioxidant vitamins, which could scavenge free radicals generated by cyclophosphamide. The increased concentration of vitamin A following ASEDS treatment shows its ability to enhance innate immunity [59], while vitamins C and E can modulate both cellular and humoral immune responses [60, 61].

Flavonoids are known for their antioxidant activities via scavenging of free radicals and upregulation of antioxidant defense systems. Their immunomodulatory roles in living systems are also reported [62]. Sinapic acid found in ASEDS has been pharmacologically evaluated for its potent anti-cancer, antioxidant, anti-inflammatory, anxiolytic, hepatoprotective, cardioprotective, renoprotective, neuroprotective, anti-diabetic, and anti-bacterial activities [63]. A study also indicated the immuno-stimulatory activity of chlorogenic acid, also identified in HPLC, by enhancing the activity of human lymphocyte proliferation and secretion of IFN-gamma [64]. A recent study by Sunil et al. [65], indicated the immunomodulatory potential of catechin-rich butanol fraction of *Acacia catechu* L. Catechins were found in the highest concentration in ASEDS from HPLC analysis (Table 3, Fig. 2).

Cyclophosphamide induction led to reduction in the levels of HDL and LDL of the negative control (Fig. 10). Treatment with ASEDS led to improvements in altered lipid levels. Group 4 rats recorded significant (*p* < 0.05) increase in HDL concentration relative to the negative control and other test groups while Group 6 rats recorded significant reduction in LDL levels. This implies that the integrity of the lipid layer of the immune cell membrane is being varied as cholesterol component is being removed and added respectively by HDL and LDL as known transporters of cholesterol in animal body. Consequently, antigen presentation on the major Histocompatibility (MHC) II of the antigen presenting cells (APCs) and subsequent recognition by appropriate T-cells would be affected because lipid constitute a greater part of MHC, suggesting that ASEDS can modulate both cellular and adaptive responses [66]. It was also observed that HDL-C concentration in each group was significantly higher than the corresponding LDL-C concentration indicating the extract possesses athero-protective capacity. The results corroborate the findings of Ogunmoyole et al. [26] who revealed that the HDL-cholesterol and LDL-cholesterol significantly increased in serum, liver and heart homogenates after administration of aqueous extract of *D. stramonium* seeds.

Several saponins isolated from medicinal plants, have been discovered to possess significant immunomodulatory effects [67], and have also shown stimulatory effects on the components of specific immunity and on monocyte proliferation. [68, 69]. The saponin, Formosanin C (Table 5), a diosgenin identified in ASEDS is known to be a crucial immunomodulatory agent [70]. Triterpenoid saponins from eight *Cephalaria* species were shown to have immunomodulatory activity [71].

ASEDS indicated presence of Epicatechin (Table 6) which have been isolated and purified from the ethyl acetate fraction of *Litchi chinensis* Sonn by reverse-phase HPLC, and was shown to have strong immunostimulatory effect proliferation of mouse splenocytes [72]. Cyanidins identified also in ASEDS (Table 6) are known to have strong antioxidant and radical-scavenging activities [73]. *Caulerpa lentillifera extract* rich in gallic acid, catechin, tannic acid, rutin, isoquercetin, and quercetin stimulated the immune response by modulating the cell cycle regulators p27, p53, cyclin D2, and cyclin E2 [74].

Sandner et al. [75] highlighted some immunomodulatory activities of Eucalyptus essential oils (EEOs). These oils include; α-Pinene, 3-Carene, Limonene, α-terpineol were identified in ASEDS (Table 7, Fig. 6). These terpenoids have been found to stimulate the immune system by increasing the amount of circulating lymphocytes and enhancing their phagocytic activity, thus improving bacterial clearance [76]. They have also been reported to suppress responses involved in inflammation and decrease cytokine production by interfering with key mediators of inflammatory pathways [77].

14. Sparge S.G., Light M.E. and Staden J.V. (2004) Journal of.

## Conclusions

This study confirmed the immunomodulatory effects of *D. stramonium L.* From the results obtained, ASEDS showed potent immunomodulatory effect which could be attributed to the presence of phytochemical constituents such as alkaloids, flavonoids, terpenoids and saponins which played key roles in normalizing altered antioxidant enzyme activities and vitamins levels as well as restoring alterations in lipoproteins levels, and stimulation of leukocytosis and increase in antibody synthesis, thus, serving as a potential candidate immunomodulatory agent.

## Materials and methods

### Materials

#### Procurement, identification and authentication of plant material

**Fig. 11:**
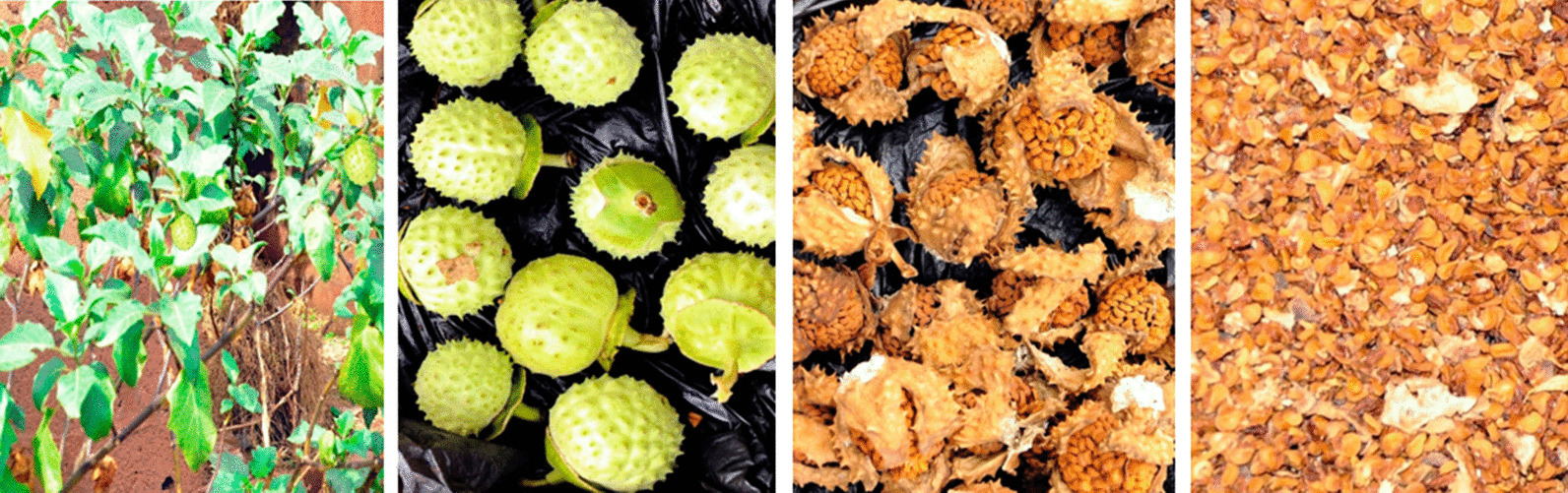
*Datura stramonium L.* plant, fruits and seeds

Approval was granted by the appropriate council for collection of the plant material. The plant material was collected in accordance with institutional best practices and as recommended by the IUCN Policy Statement on Research Involving Species at Risk of Extinction and the Convention on the Trade in Endangered Species of Wild Fauna and Flora. Ripe seeds of *Datura stramonium* L. (Solanaceae) (Fig. 11), were collected from the plant habitat in Anyigba, Dekina Local Government Area, of Kogi State, North Central Nigeria, in July 2018. The plant sample was identified by Prof. M. Adukwu, of the Herbarium of the Department of Botany, Faculty of Agriculture, Kogi State University, Anyigba, Kogi State, Nigeria, with Herbarium number, PT-121. The identity and authenticity of the plant was also confirmed with the exact sample deposited in online databases of http://www.theplantlist.org/ and http://www.ipni.org/.

#### Preparation and processing

Seeds of ripe *D. stramonium L.* were carefully separated from the ripe plant fruits, freed from sand and debris, and air-dried to a constant weight. The dried seeds were pulverized into powdered form, stored in airtight bags prior to aqueous crude extraction.

#### Study rodents

A total of Thirty (30) healthy adult Wistar rats of both sexes (180–200 g), were procured from the Animal House of the Faculty of Basic Medical Sciences, College of Health Sciences, Kogi State University, Anyigba, and were be kept in well ventilated laboratory cages. They were acclimatized to the laboratory environment for a period of seven days under standard environmental conditions, with a 12 h light/dark cycle. The animals were fed with standard feed pellets and drinking water ad libitum, and were accorded humane care throughout the period of the experiment, in line with the regulations and ethical approval of the Ethics and Biosafety Committee of the Faculty of Biological Sciences, University of Nigeria, with Reference No. UNN/FBS/EC/1046, and in accordance with the International ethical guidelines for care and use of laboratory animals [78]. The study is reported in accordance with ARRIVE guidelines available at https://arriveguidelines.org.

#### Drugs, chemicals and reagents

All chemicals and reagents used for the conduct of this study were of analytical grade. Drugs used were purchased from reputable pharmaceutical outlets in Lokoja, Kogi State, Nigeria. Freshly prepared phosphate buffer at pH 7.4 and normal saline were used for the study. Distilled and deionized water were obtained from the National Centre for Energy Research and Development (NCERD), University of Nigeria, Nsukka. Hydrogen peroxide (Interstate Chemical Co. Hermitage, PA), Bovine serum albumin (BioClot GmbH), Phosphotungstic acid (CDH Fine Chemicals, India). Orthophosphoric acid (Prime Chemicals, Gujarat, India). Ascorbic acid, oxalic acid, batophenanthroline, sodium hydroxide, iron (III) chloride, petroleum ether and ethyl acetate (Sigma Aldrich, St. Louis, MO, U.S.A.). Chloroform, methanol, ethanol, sulfuric acid, and hydrochloric acid (British Drug House, England). Fehling’s solutions A and B (Sisco Research Lab., India). Xylene (Shantou, Guangdong, China). Cyclophosphamide was purchased from Cadila Healthcare Limited, Marketed by Zydus Oncosciences, Baxter Oncology, Frankfurt Germany. Levamisole (47.3 mg) was purchased from Ecomed Pharma Limited, Ogun State Nigeria. Glutathione peroxidase (GP_X_) activity assay kit was obtained ‘Ready to use’ from Cayman Chemical, Ann Arbor, Michigan, USA. The kits for High-density lipoprotein (HDL) and Low-density lipoprotein (LDL) were products of Prestige diagnostics, Geigorim Co Antrim, United Kingdom*.* Catalase (CAT) and superoxide dismutase (SOD) assay reagent kits were procured from Randox Laboratories Ltd., United Kingdom. Immunoglobulins A, G, and M ‘Ready to use’ assay kits were procured from Weiner’s Laboratory, California, U.S.A.

### Methods

#### Aqueous extraction

Crude plant extraction was carried out according to the method of Sofowara [79] with slight modifications. A known weight, 257.6 g of the powdered seeds were soaked in 2567 mL liters of distilled water and allowed to stand for 48 h at room temperature. The mixture was then filtered using a vacuum pump, and the filtrate was lyophilized to yield 20.35 g of the aqueous seed extract of *D. Stramonium L.* (ASEDS). The percentage yield of ASEDS was determined using the formula given below.

$$\mathrm{Percentage yield }=\frac{\mathrm{weight of extract }(\mathrm{g})}{\mathrm{weight of powdered seed}(\mathrm{g})}\times \frac{100}{1}$$

#### Phytochemical analyses

Preliminary phytochemical screening of ASEDS was carried out using the methods of Harborne [80], and Pearson [81].

#### HPLC–UV analysis of ASED

High performance liquid chromatography (HPLC) was performed to identify and quantify different phytoconstituents in ASEDS. Hangzhou LC-8518 with a low-pressure gradient and solvent delivery pump with a high-pressure switching valve, a high-sensitivity ultraviolet (UV) detector (diode array), a micro syringe for sample injection, with column size of 150 × 4.6 mm, was used for the analysis.

*Sample Preparation and Extraction: *A known weight, 0.1 g of ASEDS was dissolved in 10 ml of 70% methanol and is allowed to stand for 1 to 2 h in a closed test tube. The extracted sample is then decanted, centrifuged and filtered using a cosmonice filter or micron filter into a 5 ml sample bottle.

*Preparation of Standard Solutions: *Stock solutions of the reference compounds were made by weighing 0.001 g of reference standards into a test tube and dissolving each standard with 10 ml of 70% methanol. Each standard was then agitated for 10 min using vortex mixer and then filtered using a cosmonice filter or micron filter into the sample bottle.

- Determination of alkaloids

*Chemicals:* Methanol (lichrosolv), acetonitrile (lichrosolv), nicotine, anatabine, cotinine, anabasine, and myosmine (analytical grades) reference standard.

*Procedure:* The mobile phase comprised of methanol/acetonitrile/water (70:20:10), with wavelength set at 260 nm. Column temperature was set to 40 °C, with run time set at 15 min. ASEDA volume of 40 µl was injected. The mobile phase was pumped-in, transferring the sample into the column. The chromatogram was obtained from the display system after the run time. The retention time of the standard is compared to that of the chromatogram obtained from the sample to determine the content of alkaloids in ASEDS.

(b) Determination of flavonoids

*Chemicals:* Formic acid (analytical grade), methanol (lichrosolv), acetonitrile (lichrosolv), analytical grades of rutin (95%), quercetin (95%), quercitrin (85%), kaempferol (90%) and isorhamnetin (99%) reference standards.

*Procedure:* Mobile phase, acetonitrile/water/formic acid (25:74:1) was prepared. The wavelength was set at 254 nm, with column temperature set to 40 °C. Run time was adjusted to 25 min. Sample volume of 40 µl was injected. The mobile phase was pumped-in which allowed the sample to be transferred into the column. The total ion chromatogram is obtained from the display system after the run time. The retention time of the standard was compared with that of the chromatogram obtained from the sample to determine the flavonoid content of ASEDS.

(iii) Determination of phenolics

*Chemicals:* Glacial acetic acid (analytical grade), methanol (lichrosolv), acetonitrile (lichrosolv), analytical grades of ascorbic acid, gallic acid, catechin, methy gallate, caffeic acid, syringic acid, ellagic acid, chlorogenic acid reference standards.

*Procedure*: Mobile phase was prepared by mixing acetonitrile, water and acetic acid (19:80:1). The wavelength was set at 272 nm, while column temperature was set to 40 °C. Run time is set at 25 min. Sample volume of 40 µl was injected. The mobile phase was pumped-in into the column. The chromatogram was obtained from the display system after the run time. The retention time of the standard was compared with that of the chromatogram obtained from the sample to determine the content of phenolic compounds in ASEDS.

(iv) Determination of saponins

*Chemicals:* Methanol (lichrosolv), acetonitrile (lichrosolv), diosgenin, gitogenin or hecogenin (analytical grades) reference standard.

*Procedure:* The mobile phase was prepared by mixing acetonitrile and water in the ratio of 70:30. The wavelength was set at 205 nm, with column temperature maintained at 40 °C. Run time was set at 14 min. A sample volume of 40 µl was injected, and the mobile phase was pumped-in which moved the sample into the column. The HPLC chromatogram was obtained from the display system after the run time. The retention time of the standard is compared with that of the chromatogram obtained for ASEDS to determine the saponin content.

(e) Determination of tannins

*Chemicals:* Methanol (lichrosolv), Tannic acid (analytical grade) reference standard.

*Procedure*: The mobile phase, methanol/water (50:50) was prepared. Wavelength was adjusted to 270 nm. Column temperature was kept at 40 °C. Run time was 6 min. Sample volume of 40 µl was injected into the instrument. The mobile phase transferred the sample into the column for analysis. The chromatogram obtained from the display system after the run time was compared with that of the standard to determine the tannin content of ASEDS.

(f) Determination of terpenoid

*Chemicals:* Formic acid (analytical grade), Methanol (lichrosolv), corosolic acid, betulin, betulinic acid, oleonolic acid, ursolic acid (analytical grades) reference standards.

*Procedure:* The mobile phase was prepared, methanol/water/formic acid in the ratio (90:9.9:0.1). Wavelength was set at 210 nm, with column temperature adjusted to 40 40 °C. The run time was set at 25 min. A sample volume of 40 µl was injected. The mobile phase is pump in which allowed the sample to be carried into the column. The chromatogram was obtained from the display system at end of experiment. The retention time of the standard was compared with that of the chromatogram obtained from ASEDS to determine its terpenoid content.

#### Induction of immunosuppression

Immunosuppression in Wistar rats was induced according to the protocol of Joshua et al. [27]. Accordingly, 10 mg/kg b.w. of Cyclophosphamide was administered orally for 27 days to induce immunosuppression in designated rat groups.

#### Experimental design

The experimental design of Kyakulaga et al. [82] as modified, was adopted. Thirty (30) rats were divided into six groups of 5 rats each. Group 1 served as normal control and received 1 mL/kg b.w. distilled water throughout the duration of the experiment. Group 2 served as negative control and was pretreated with 10 mg/kg b.w. Cyclophosphamide orally for 27 days followed by 1 mL/kg b.w. distilled water given orally for 28 days’ post treatment. Group 3 served as standard control, and were pretreated with 10 mg/kg b.w. Cyclophosphamide followed by 5 mg/kg b.w. of Levamisole [44]. Groups 4, 5 and 6 made up the ASEDS test groups. Rats in these groups were orally pre-treated with 10 mg/kg b.w. Cyclophosphamide for 27 days, followed by 60, 90, and 120 mg/kg b.w. of ASEDS respectively [83], administered orally for 28 days. Thereafter, the rats were fasted overnight, and euthanized by cervical dislocation prior to sacrifice on day 29 [84]. Fresh blood samples were collected via cardiac puncture and emptied into neatly labeled plain sample tubes for biochemical analysis, and EDTA-containing sample tubes for leukocyte count and differentials. The blood in plain tubes were subjected to centrifugation at 3000 rpm for 10 min. Serum obtained were stored in the refrigerator for subsequent biochemical analysis.

#### Determination of biochemical indices

Total leucocytes and differential cell counts were determined using an automated hematology analyzer (URIT-330) based on Coulter’s method as described by Robinson [85] Serum catalase activity was assayed following to the method of Sinha [86]. Superoxide dismutase activity was assayed by the inhibiting of auto-oxidation of epinephrine, using the protocol of Pajovic et al. [87]. Glutathione peroxidase activity was assayed according to the method of Paglia and Valentine [88] as described by Ekpo et al. [84]. The concentration of serum Vitamin A was determined by the method of Rutkowski et al. [89]. Serum vitamin C concentration was determined using the method of Rutkowski et al. [90], while serum vitamin E concentration was determined according to the method of Rutkowski et al. [91]. High density lipoprotein concentration in serum was determined according to the method described by Kameswara et al. [92], while Low-density lipoprotein concentration was determined by the method of Arsman et al. [93], using an Auto-analyzer (URIT-810).

#### Determination of serum immunoglobulin A, G and M concentrations

Serum immunoglobulin A, G, and M concentrations were determined according to the method of Pressac et al. [94]. The method is based on the principle that immunoglobulin reacts with specific antigen to generate insoluble immune complexes. The turbidity of the complexes is directly proportional to the immunoglobulin concentration in the sample which can be measured using a spectrophotometer. Briefly, a calibration curve was drawn from serial dilutions of the calibrator protein in saline solution at 1:10, 1:20, 1:40, 1:80, and 1:160 using saline solution as zero point. 40 µL of diluted calibrator protein was mixed with 900 µL of Reagent A (buffered saline solution). The mixture was homogenized and the absorbance of the dilutions were measured at 340 nm as OD_1,_ after calibrating the instrument to zero with distilled water as blank. An aliquot of 160 µL of Reagent B (antibody monospecific anti-IgA, IgG, or IgM) was then added to the mixture and incubated at room temperature for 30 min. The absorbance of the reacting mixture (OD_2_) was measured against the blank. The difference in absorbance (OD_2_–OD_1_) for each calibrator protein dilution, including the zero point was calculated. A plot of calibrator protein concentrations in mg/dl against the differences in absorbance was plotted. For the sample, an aliquot of 10 µL was mixed with 100 µL of saline solution to achieve a 1:10 dilution. Thereafter, 40 µL of the diluted sample was mixed with 900 µL of Reagent A. The mixture was homogenized and the absorbance of the dilution was measured at 340 nm as OD_1_ using distilled water as blank. Thereafter, 160 µL of Reagent B was then added to the mixture and incubated at room temperature for 30 min. The absorbance of the reaction mixture (OD_2_) was measured against the blank. The difference in absorbance was determined, and the concentration of immunoglobulin A, G, and M respectively, in the serum (mg/dl) was determined from the standard calibration plot.

#### Statistical analyses

The data obtained from the study were analyzed using IBM Statistical Product and Service Solutions (SPSS) version 21.0 (Chicago, IL), and GraphPad Prism version 7.0. Significant differences in the means were established by the one-way analysis of variance (ANOVA) using the Dunett’s post hoc multiple comparison test). The results were presented as mean ± standard deviation of replicate measurements. Mean values with *(*p* ≤ 0.05) were significantly different.

## Data Availability

More data used and/or analysed during the current study are available from the corresponding author on reasonable request.

## Abbreviations

ASEDS: Aqueous seed extract of *Datura **stramoniu**m*
HDL: High-density lipoprotein
LDL: Low-density lipoprotein
HPLC: High-performance liquid chromatography
CAT: Catalase
SOD: Superoxide dismutase
GPx: Glutathione peroxidase
CSF: Colony stimulating factor
WHO: World Health Organization
